# PReS-FINAL-2223: The relationship between FMF, BD and epilepsy

**DOI:** 10.1186/1546-0096-11-S2-P213

**Published:** 2013-12-05

**Authors:** H Sargsyan

**Affiliations:** 1 Yerevan State Medical University, Yerevan, Armenia

## Introduction

Familial Mediterranean Fever(FMF) is an autoinflammatory disease particularly frequent around the Mediterranean basin. Behçet disease (BD) is a autoinflammatory disease which distribution is mainly around the Mediterranean Sea, Middle East and Far East.Epilepsy is a chronic disorder of the brain that affects people in every country of the world.It is characterized by recurrent seizures.

## Objectives

Coexistence of FMF with BD and also association FMF with epilepsy has been reported in some studies.Here we present a case of FMF, BD and epilepsy in the same family.

## Methods

Clinical and laboratory findings are presented.

## Results

7 year old boy admitted to the Arabkir Centre with complains of: recurrent abdominal pain, fever, oral ulcers, recurrent genital ulcerations with skin lesions.Manifestation of disease began from one year with fever, recurrent oral ulcerations 8 times in 12 months. From 2 years began abdominal pain, chest pain.The attacks was 3 times per month each lasting typically for 3 days.The family history was: father had epilepsy with FMF, MEFV mutations was: M694/F479L. He received colchicine and antiepileptic drugs.Sister of the father had recurrent abdominal pain, fever, arthritis, oral ulcers, skin lesions(she was not in Armenia). Examinations; FBC results showed leucocytosis with elevated CRP and ESR. Chest X-ray revealed exudates in the costophrenic angle from the two sites.Genetic investigation of MEFV reviled M694V/N heterozygotes mutations.  HLA*B51 was positive. We established the diagnosis of FMF according criteria Tel-Hashomer and BD: according criteria for Behçet's disease, rule out other conditions with similar symptoms.It was started the treatment with colchicines 1 mg/day. The attacks of FMF was resolved but oral ulcerations with skin and genital problems was continued. After the prednisone therapy with the colchicine treatment the problem was resoled.

## Conclusion

This is an interesting case presenting probable relationship between FMF, BD and epilepsy. We suppose, that these diseases are connected not only with the relationship of geneses, but also with major pathological development in mesenchymal tissue in morphogenesis.

## Disclosure of interest

None declared.

